# Study on the Axial Compressive Behavior of Steel Fiber Reinforced Concrete Confined with High-Strength Rectangular Spiral Stirrup

**DOI:** 10.3390/ma18030669

**Published:** 2025-02-03

**Authors:** Huajing Zhao, Weitong Liu, Penghui Yang, Can Song

**Affiliations:** 1 School of Science, Xi’an University of Architecture and Technology, Xi’an 710055, China; 2 General Institute of Design and Research, Xi’an University of Architecture and Technology, Xi’an 710055, China; yph_003@163.com; 3 Xi’an Wuhe New Materials Technology Group Co., Ltd., Xi’an 710055, China; brightscan@126.com

**Keywords:** high-strength rectangular spiral stirrup, steel fiber reinforced concrete, lateral binding force, stirrup stress, constitutive relation

## Abstract

Monotonic axial compression tests were carried out on 16 steel fiber-reinforced concrete (SFRC) columns confined by rectangular spiral stirrups. The impacts of stirrup spacing, stirrup strength, concrete strength, and cross-sectional aspect ratio on the peak load, ductility, and failure mode of these columns were analyzed. The test results demonstrate that steel fibers significantly mitigate the spalling of the concrete column’s protective layer through their bridging effect. Small spacing and high-strength spiral stirrups effectively confine the core concrete, enhancing the bearing capacity and ductility of concrete columns. Concrete strength exhibits a positive correlation with the confinement effect. However, as concrete strength increases, the rate of improvement in the confinement effect decreases. At peak compressive stress, the high-strength stirrup may not reach its yield state. Based on the test results, a method for calculating stirrup stress under the peak stress of confined concrete is proposed. A “coupling restraint coefficient” is proposed, and a constitutive model for HRSS confined steel fiber reinforced concrete is developed, considering the coupled effect of effective confinement forces in different directions. Comparative analysis shows that the constitutive model established in this paper agrees well with the experimental results and demonstrates good applicability.

## 1. Introduction

As building structures increasingly demand high strength, durability, and seismic performance, Steel Fiber Reinforced Concrete (SFRC) has emerged as a promising composite material. It offers significant advantages in enhancing tensile strength, cracking resistance, ductility, and toughness. SFRC has found widespread applications in bridges, tunnels, high-rise buildings, industrial floors, and other areas. The inclusion of steel fibers effectively disperses stress and bridges cracks, thereby improving the mechanical properties of concrete. Particularly under axial loading, steel fibers can delay crack propagation, improving the ductility and damage resistance toughness of concrete [1,2,3]. However, the axial compression performance of SFRC is still influenced by factors such as concrete strength, stirrup strength, stirrup spacing and configuration, and the reinforcement ratio of longitudinal bars. Improving its axial load-bearing capacity and ductility remains a key focus of ongoing research.

Relevant studies have demonstrated that the lateral restraint provided by spiral stirrups can effectively prevent the lateral expansion of concrete under axial load, thereby reducing crack propagation. This significantly enhances the compressive strength and deformation capacity of concrete [4,5,6]. Iyengar et al. [7] introduced the concept of the “constraint coefficient” to quantify the influence of stirrups on the compressive strength and deformation capacity of concrete. Sheikh et al. [8] proposed the “effective constrained area” concept, which accounts for the stirrup’s restraint effect in rectangular cross-section columns. Their research shows that the passive binding force exerted by stirrups on concrete is non-uniformly distributed and influenced by the “arch effect.” Mander et al. [9,10] improved the calculation method for the “effective confinement area,” developed a unified stress–strain model applicable to circular, square, and rectangular cross-sections, and introduced the “effective confinement coefficient,” which incorporates the stirrup’s influence on concrete’s lateral confinement. Building on this, Cusson et al. [11,12] developed a stress–strain model for confined high-strength concrete. They emphasized that the actual stress of the stirrup at peak column strength should be used, rather than the yield stress, in calculating effective confined stress. Legeron [13] proposed an iterative method for calculating stirrup stress when confined concrete reaches compressive strength. However, it is important to note that the formula assumes steel bars behave as ideal elastic–plastic materials, which may not be applicable to high-strength steel bars. Although numerous studies have established full-curve models for axial compression performance in stirrup-constrained ordinary concrete columns, there is a lack of research on high-strength spiral stirrup-constrained lightweight aggregate concrete columns. Further investigation into their mechanical behavior and design models is urgently needed.

Khaloo et al. [14] conducted experiments on high-strength lightweight aggregate concrete columns confined by circular spiral stirrups with complex cross-sections. They proposed a calculation model for peak stress and peak strain and developed a corresponding constitutive model that accounts for the influence of multiple factors. Shin et al. [15] tested nine stirrup-confined lightweight aggregate concrete columns, examining the effects of hybrid fibers, stirrup configuration, spacing, and diameter on the columns’ performance. Ding et al. [16] performed axial compression tests on 24 high-strength stirrup-confined lightweight aggregate concrete columns, finding that reducing stirrup spacing or using composite stirrups improves both the bearing capacity and ductility of these columns. Based on these axial compression tests, researchers worldwide have developed various uniaxial compression constitutive models [17,18,19,20]. The high-strength rectangular spiral stirrups effectively restrain the lateral expansion of the concrete. This not only improves the compressive strength of the component but also enhances its bending and shear responses. As a result, the overall load-bearing capacity and shear resistance of the reinforced concrete component are increased [21,22,23]. Therefore, high-strength rectangular spiral stirrups are applied to steel fiber-reinforced concrete columns in this study, aiming to investigate their axial compressive behavior and constitutive model under high-strength confinement.

Through experimental studies, scholars have revealed several factors that influence the performance of confined concrete and have proposed methods for calculating the compressive strength, peak compressive strain, and stress–strain relationship models of confined concrete. However, several issues remain to be addressed in this field. Many studies overlook the possibility that stirrups may not yield under the peak compressive stress of confined concrete. While some studies have addressed this, the methods for calculating stirrup strain are often complex, or the results show significant deviations from experimental data. Additionally, research on steel fiber-reinforced concrete columns confined by rectangular spiral stirrups with different cross-sectional aspect ratios is still limited.

The research shows that with an increase in the steel fiber content, the compressive strength of concrete slightly improves. However, for low dosages of steel fibers (e.g., 1%), the increase in compressive strength is relatively small [24]. Therefore, this paper focuses on experimentally studying the confinement effect of high-strength rectangular spiral stirrups on steel fiber-reinforced concrete columns and systematically investigates the failure modes and mechanical behaviors of the columns. The study primarily analyzes the influence of factors such as cross-sectional aspect ratio, concrete strength, stirrup strength, and stirrup spacing on axial load-bearing capacity and ductility. A substantial amount of experimental data on stirrup-yielding and non-yielding behavior under peak compressive stress was obtained. Based on these findings, an expression for the tensile stress in the stirrup when the confined concrete reaches its compressive strength is proposed, and a new constitutive model is proposed.

These research findings not only provide new theoretical insights for the design of SFRC columns but also offer valuable references for the practical design of building structures. The use of high-strength rectangular spiral stirrups significantly enhances the compressive strength and ductility of structures, making it especially beneficial in earthquake-prone areas and under high-load conditions. Therefore, the results of this study can offer theoretical support and engineering guidance for the seismic design of related structures.

## 2. Test Profile

### 2.1. Specimen Design

**Figure 1:**
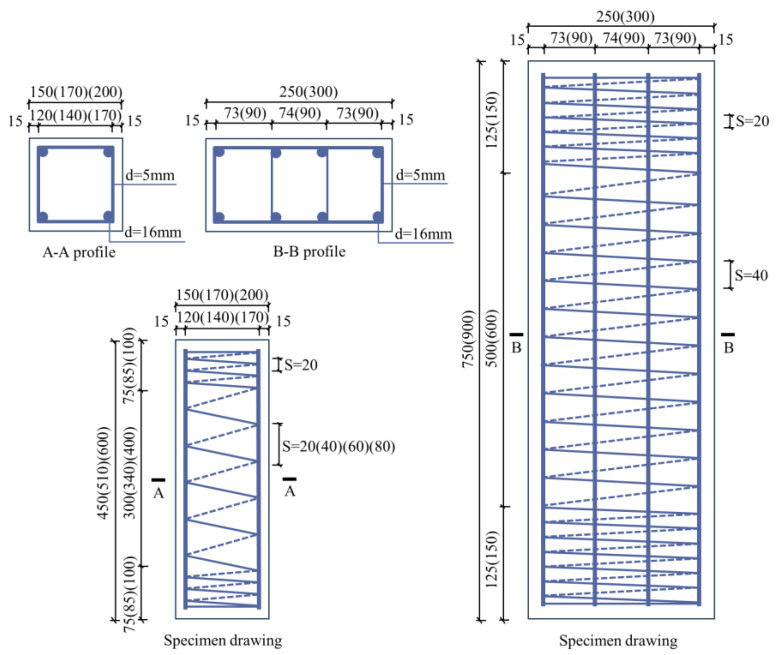
Specimen size and reinforcement diagram (unit: mm).

Monotonic axial compression tests were conducted on 16 steel fiber-reinforced concrete columns confined by high-strength rectangular spiral stirrups, as presented in this study. The main design parameters of the specimens, including section size, concrete strength, stirrup strength, and stirrup spacing, are listed in Table 1. The thickness of the column protective layer is 15 mm, with a stirrup-encrypted zone featuring a 20 mm spacing at 1/6 of the end to prevent local damage. The middle two-thirds of each specimen was used for testing. The reinforcement and cross-section details of the specimens are shown in Figure 1.

### 2.2. Properties of Raw Materials

#### 2.2.1. Concrete

**Figure 2:**
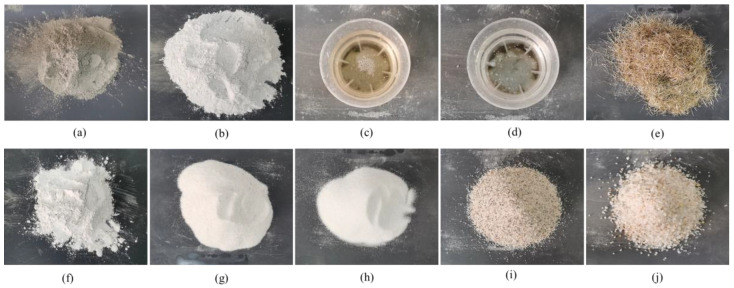
Raw materials used in SFRC. (**a**) P.O 42.5R cement; (**b**) silica fume; (**c**) water reducing agent; (**d**) defoamer; (**e**) steel fiber; (**f**) 325 mesh quartz sand; (**g**) 70–140 mesh quartz sand; (**h**) 40–70 mesh quartz sand; (**i**) 20–40 mesh quartz sand; (**j**) 10–20 mesh quartz sand.

**Figure 3:**
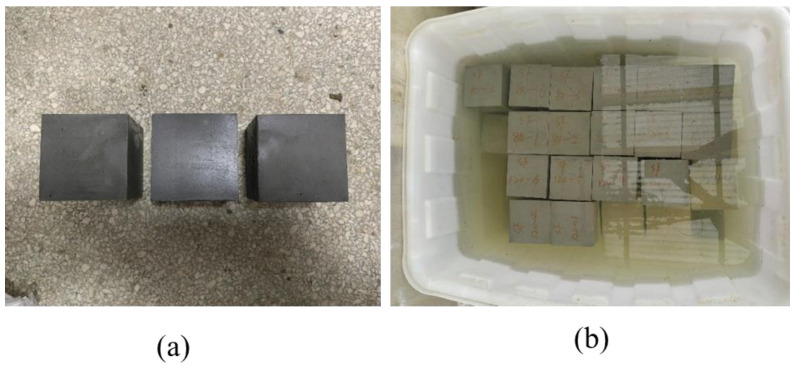
Cube test block making. (**a**) Test block making; (**b**) test block curing.

In the preparation of SFRC, P.O 42.5R ordinary Portland cement, silica ash (enhance the density, strength, and durability of concrete), polycarboxylic acid high-performance water reducer, cement gypsum antifoam agent, 14 mm copper-coated steel fiber, and quartz sand were selected, as shown in Figure 2. Quartz sand includes 10–20 mesh, 20–40 mesh, 40–70 mesh, 70–120 mesh, and 325 mesh five particle sizes, the mass ratio of 1.1:0.6:1.5:1.3:1.4. The design mix ratio is shown in Table 2. The steel fibers are copper-coated to enhance their corrosion resistance and crack resistance, with performance indicators shown in Table 3. As shown in Figure 3, Three standard cubic test blocks were poured for each grade of SFRC to measure the compressive strength (*f*_cu_) and obtain an average value. The results of basic mechanical properties are listed in Table 4.

#### 2.2.2. Rebar

In order to measure the properties of rebar with different strengths, samples were prepared and tested according to Chinese standard GB/T 228.1-2010 [25]. The key parameters of yield stress (*f*_y_), elastic modulus (*E*_s_), and yield strain (*ε*_y_) were calculated according to the stress–strain curves of each sample recorded during the test. The specific data are shown in Table 5.

### 2.3. Specimen Preparation

**Figure 4:**
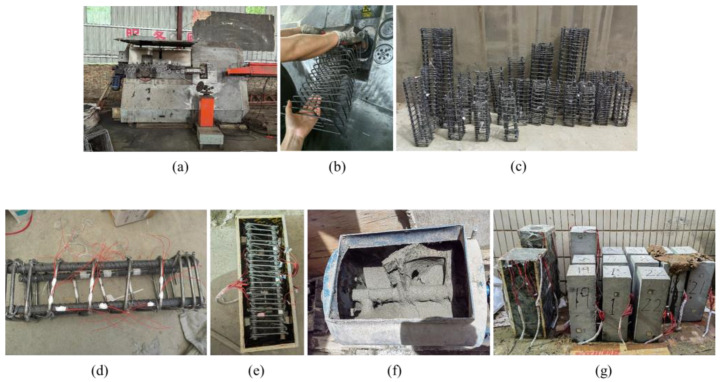
Specimen making. (**a**) Automatic hoop bending machine device; (**b**) spiral stirrup production; (**c**) steel cage making; (**d**) attach steel bar strain gauge; (**e**) support form; (**f**) concrete mixing; (**g**) specimen curing.

For the production of HRSS confined steel fiber reinforced concrete columns, the longitudinal steel bars are shortened by 20 mm compared to the test pieces to ensure they are not directly compressed and only interact with the concrete along with the stirrups. The production process, as shown in Figure 4, includes the following steps: First, the spiral stirrups are fabricated using an automatic hoop bending mechanism, and the longitudinal reinforcement is bound to form a steel cage. Next, the steel bars are polished and cleaned, followed by the application of 502 paste strain gauges. Silicone rubber 703 is applied, and the raw material belt and transparent tape are used for waterproofing. The steel cage is then placed in a wooden mold coated with a release agent. The SFRC is poured and vibrated using a shaking table to ensure uniform density. After fabrication, the sample is covered with a plastic film and left in the mold for 24 h in a laboratory environment at 20 ± 2 °C and 60 ± 5% relative humidity. Following this, the sample is carefully unmolded and cured in a standard curing chamber at 20 ± 2 °C and 95% humidity until testing.

### 2.4. Loading Device and Loading System

**Figure 5:**
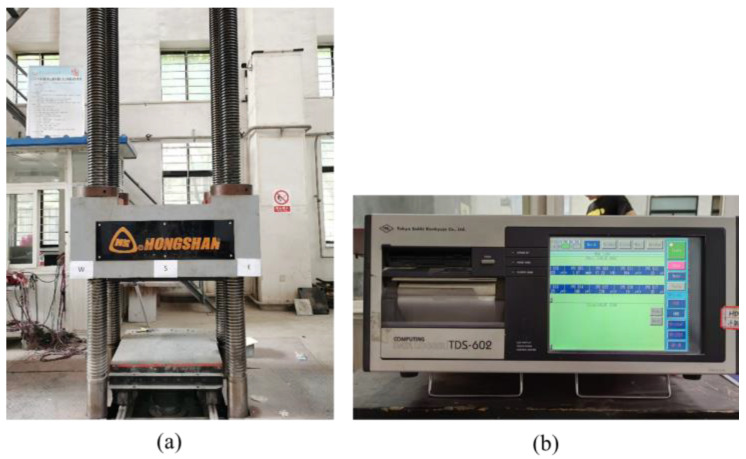
Test loading device. (**a**) YAW-5000 microcomputer controlled electro-hydraulic servo pressure testing machine; (**b**) TDS-602 Static data acquisition instrument.

The test was carried out in the Key Laboratory of Structure and Earthquake Resistance of the Ministry of Education, Xi’an University of Architecture and Science and Technology. A 5000 kN hydraulic testing machine was used to carry out the axial compression test. The test loading device is shown in Figure 5a.

To ensure uniform load application, a thick layer of fine sand was placed at both the upper and lower ends of the specimen for leveling. During the formal loading process, displacement control mode was employed with a loading rate of 0.2 mm/min, which was maintained constant throughout the test. The loading was stopped when the axial load of the concrete column decreased to 65% of the peak load. During the test, the load values, as well as the readings from the strain gauges and displacement meters, were automatically collected using the TDS-602 static data acquisition system, as shown in Figure 5b.

### 2.5. Test Content and Test Point Layout

**Figure 6:**
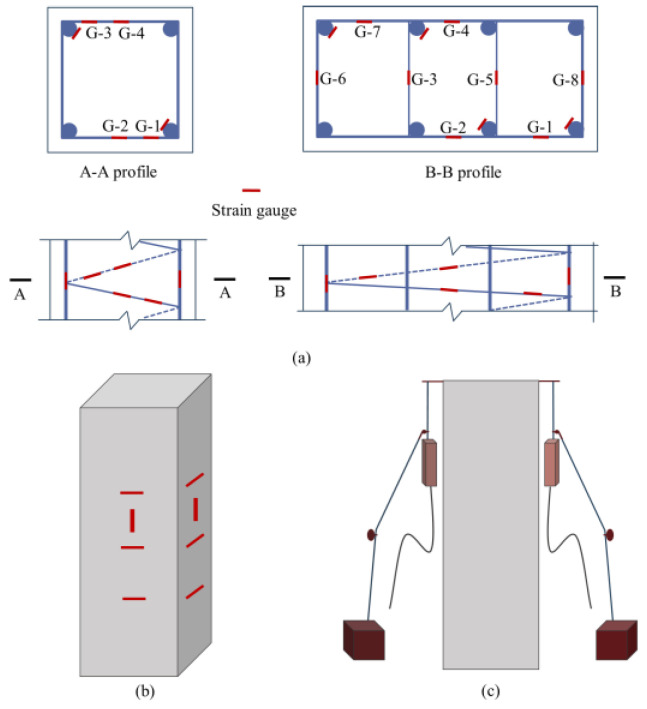
Strain gauge and displacement gauge layout. (**a**) Intermediate bar strain gauge layout; (**b**) concrete strain gauge layout; (**c**) displacement meter layout.

**Figure 7:**
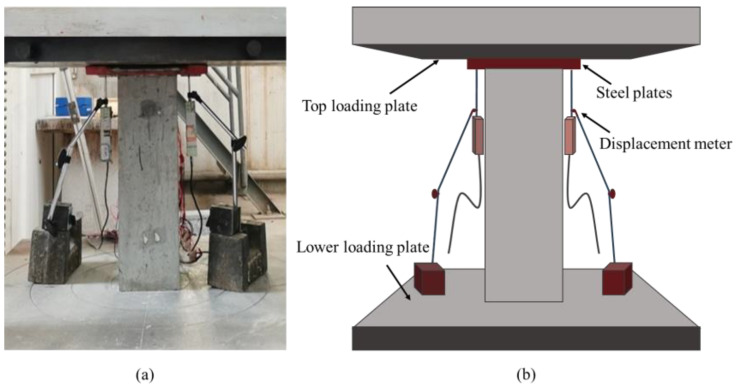
Loading device diagram. (**a**) Test device drawing; (**b**) schematic diagram of the test device.

The main measurement parameters in the test include axial load, longitudinal and transverse displacement, as well as stirrup and longitudinal strain. As shown in Figure 6a, resistance strain gauges were affixed to the three coils of the spiral stirrup in the test section. Four strain gauges were placed on each loop of the rectangular stirrup, and eight strain gauges were attached to the rectangular composite stirrup, labeled G-1 to G-4 and G-1 to G-8, respectively. Resistance strain gauges were also pasted onto the diagonal longitudinal bars of the steel cage, with three strain gauges arranged on each bar, marked Z-1 to Z-3 from top to bottom. As shown in Figure 6b, one longitudinal strain gauge and three transverse strain gauges were positioned at the middle of each side of the specimen to measure axial strain and transverse strain at the section. Two displacement gauges were installed between the upper and lower pressure plates of the press, as shown in Figure 6c. A schematic diagram of the loading device is presented in Figure 7.

## 3. Test Results and Analysis

### 3.1. Test Phenomenon

**Figure 8:**
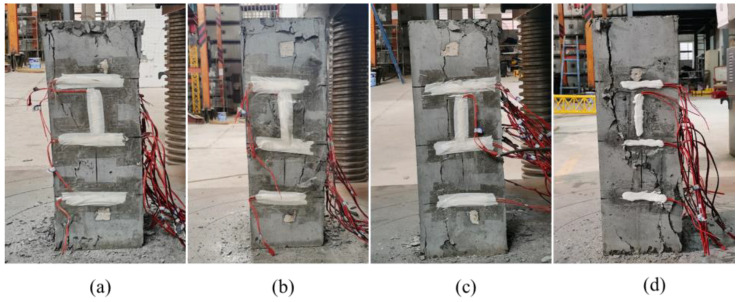
Failure phenomena of specimens C-1, C-6, C-7, and C-8. (**a**) C-1; (**b**) C-6; (**c**) C-7; (**d**) C-8.

**Figure 9:**
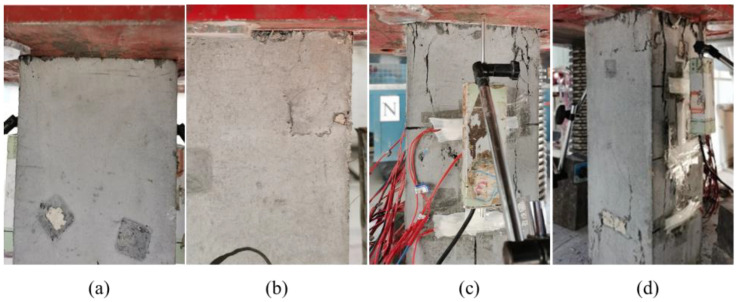
Failure phenomena of specimens at different stages. (**a**) Initial loading; (**b**) cracking time; (**c**) 80% peak load; (**d**) peak load time.

The failure process of all specimens followed a similar pattern, exhibiting good ductility characteristics. However, as stirrup spacing increased, the cracks during failure gradually widened, and the failure modes became more pronounced. Taking typical specimens C-1, C-6, C-7, and C-8 as examples, the failure phenomena of specimens at different stages are shown in Figure 8, and the final failure is shown in Figure 9.

In the initial loading stage, the specimen experienced elastic deformation with little noticeable deformation. During this phase, a synergistic effect between the SFRC and steel bars occurred, and the restraint effect of the high-strength stirrups had not yet been fully activated, so no clear failure characteristics were observed on the specimen surface. When the load reached approximately 40% of the peak load, small vertical cracks began to appear at the corners of both ends of the specimen. The expansion of these cracks was slow and gradually stabilized. At the same time, a few small horizontal cracks started to appear on the surface of the middle section of the specimen. However, the number of cracks was limited, their widths remained small, and the crack expansion was initially constrained by the HRSS.

As the load reached approximately 80% of the peak value, the specimen entered the elastoplastic deformation stage. Although the load growth rate remained steady, the deformation rate of the specimen accelerated significantly, and plastic deformation characteristics became increasingly evident. The number of horizontal cracks on the concrete’s middle surface increased rapidly, and the crack widths grew noticeably. Meanwhile, the vertical cracks at the specimen’s corners widened, accompanied by “sizzling” and “crackling” sounds as the cracks expanded. During this stage, the restraining effect of the HRSS gradually intensified, effectively inhibiting further crack propagation. However, cracks continued to expand, particularly in the middle section of the concrete.

As the load approaches the peak, the axial deformation of the specimen increases sharply, and the cracks expand and propagate rapidly. The cracks in the middle section intersect and extend throughout the entire specimen. After reaching the peak load, the specimen’s bearing capacity rapidly declines, the width of the horizontal cracks continues to increase, and the vertical main crack gradually develops. Although the transverse deformation of the specimen increases significantly, the effective restraint provided by the HRSS allows the specimen to maintain a certain level of bearing capacity. Crack expansion is constrained, and the specimen does not become completely unstable. When the load drops to about 60% of the peak load, the specimen, still supported by the HRSS, does not fully fail but instead enters a relatively stable failure stage.

Finally, the failure mode of the specimen reveals that cracks initiate from the vertical cracks at the corners of both ends and gradually spread toward the center of the specimen. As the load increases further, these cracks converge and form the main cracks that run through the entire specimen, ultimately resulting in vertical cracks. The surface of the HRSS confined steel fiber reinforced concrete column exhibits debris detachment due to crack propagation. However, unlike ordinary confined concrete columns, the protective layer does not experience detachment in the same manner. This is because the steel fiber is well-bonded to the matrix. When cracks appear in the specimen, the bridge action of the steel fiber and the restraint action of the stirrup inhibit the development of cracks and slow down the growth rate of cracks.

### 3.2. Test Result

The axial load of the specimen is shared by the concrete in the core area, the concrete protective layer, and the longitudinal reinforcement. The peak stress *f*_cc_ of the SFRC in the core area can be calculated using Equation (1):

(1) $$f_{\mathrm{cc}}=\frac{N_{u}-A_{c,\mathrm{cov}}\sigma_{c,\mathrm{cov}}\left(\varepsilon_{c,\mathrm{cov}}\right)-A_{s}\sigma_{s}\left(\varepsilon_{s}\right)}{A-A_{\mathrm{cov}}-A_{s}},$$

where *N*_u_ is the peak load of the specimen; *A*, *A*_cov_, and *A*_s_ are the cross-sectional area of the specimen, the area of the concrete protective layer, and the cross-sectional area of the longitudinal reinforcement, respectively. *σ*_c,cov_(*ε*_c,cov_) and *σ*_s_(*ε*_s_) represent the stress of the concrete protective layer and the longitudinal reinforcement, respectively.

Each load-bearing column was tested with only one specimen, and the test results for each specimen are shown in Table 6. Where *ε*_cc_ is the axial strain at peak strength, *ε*_0.85_ is the axial strain when the peak strength drops to 85%, and *ε*_0.65_ is the axial strain when the peak strength drops to 65%. The peak strength ratio (*K*_f_ = *f*_cc_/*f*_c0_) and the ductility ratio (*μ*_ε_ = *ε*_0.85_/*ε*_cc_) were used to quantify the strength improvement and ductility level of the specimens, respectively.

Based on the data in the table, the peak strength ratio *K*_f_ ranges from 1.10 to 2.03, indicating that the HRSS confinement significantly improves the compressive strength of steel fiber-reinforced concrete, surpassing that of plain concrete. The ductility ratio *μ*_ε_ ranges from 1.11 to 1.59, demonstrating that the restraining effect of HRSS notably enhances the ductility and deformation capacity of the concrete during the loading process.

### 3.3. Stress–Strain Curve Analysis

**Figure 10:**
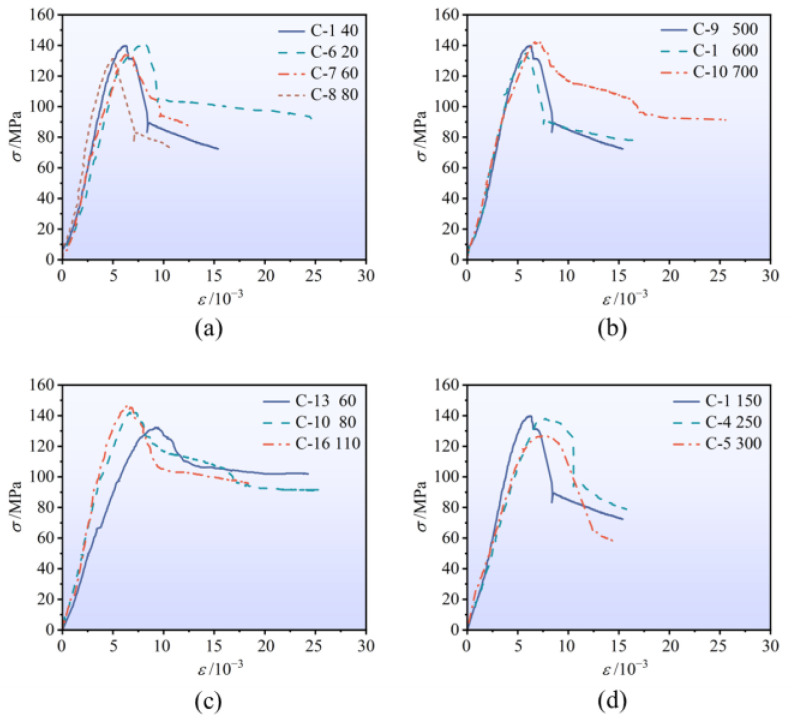
Comparison of stress–strain curves of specimens with different influencing factors. (**a**) Different stirrup spacing; (**b**) different stirrup strength; (**c**) different concrete strength; (**d**) different cross-sectional aspect ratio.

The stress–strain curves of each specimen are shown in Figure 10, where the horizontal axis represents strain, and the vertical axis represents stress. In the initial loading stage, the stress–strain curve of HRSS confined steel fiber reinforced concrete exhibits a linear relationship with a steep slope, indicating that the stress increases rapidly with axial strain. As the load approaches 80% of the peak stress, the concrete protective layer of the specimen begins to crack, causing a slowdown in the growth rate of axial stress. The slope of the curve decreases, and the HRSS-confined steel fiber reinforced concrete transitions into the elastoplastic deformation stage. As the load continues to increase, the specimen reaches its peak stress. After this point, the curve enters the descending section. Due to the further development of cracks, the stress of the specimen reduces to approximately 85% of the peak stress. At this stage, the transverse expansion and deformation of the concrete accelerate, while HRSS exerts a strong restraining effect on the concrete in the core area, thereby slowing the rate of stress decline. Consequently, the stress–strain curve of the specimen flattens. Analysis of Figure 10 reveals the influence of various factors on the bearing capacity and ductility of the specimen, which are discussed in detail below.

#### 3.3.1. Stirrup Spacing

As shown in Figure 10a, a reduction in stirrup spacing leads to a significant increase in peak stress, a slower rate of stress attenuation in the softening section, and a notable enhancement in ductility. Smaller stirrup spacing significantly improves the restraining effect on the concrete, thereby enhancing both the bearing capacity and ductility of the specimens. However, when the spacing of the stirrups is reduced to a certain extent, its effect on the peak strength weakens, while its impact on ductility becomes more pronounced. This is because, at smaller stirrup spacings, the restraining effect has reached saturation, and the presence of steel fibers partially reduces the influence of stirrup spacing on compressive strength. To balance energy conservation, emission reduction, and excellent compressive performance, it is recommended to set the stirrup spacing between one-quarter and one-third of the core concrete’s side length. This range provides a reasonable compromise between the restraint effect and material utilization.

#### 3.3.2. Stirrup Strength

The influence of stirrup strength on the stress–strain curve of confined concrete with a concrete strength of 100 MPa is illustrated in Figure 10b. It can be observed that as the stirrup strength increases, the peak strength of the specimen is significantly enhanced. Moreover, the stress attenuation rate in the softening section is notably slowed, and the ductility performance is improved.

#### 3.3.3. Concrete Strength

The effect of concrete strength on the stress–strain curve of confined concrete with a stirrup strength of 700 MPa is presented in Figure 10c. It can be observed that as the concrete strength increases, the peak stress significantly rises, but the ductility gradually decreases. Low-strength concrete shows a slower stress drop during the softening stage and a longer strain range in the residual stress stage, exhibiting better ductility and energy dissipation capacity. In contrast, while high-strength concrete significantly increases the peak stress, the stress decreases more rapidly in the softening stage, and the residual stress stage is shortened, leading to a clear reduction in ductility. This indicates that improving concrete strength significantly enhances load-bearing capacity but also increases the material’s brittleness, which can be mitigated by external confinement measures.

#### 3.3.4. Cross-Sectional Aspect Ratio

The increase in the peak stress of the column gradually decreases with the increase in the cross-sectional aspect ratio of the section, as shown in Figure 10d. This is because, with a larger cross-sectional aspect ratio, the confinement effect of the spiral stirrups on the core concrete weakens, especially in the long direction. The three-dimensional stress state of the core concrete becomes uneven, leading to a decrease in strength. Regarding ductility, a moderate increase in the cross-sectional aspect ratio improves the column’s ductility, manifested by a slower decline of the curve after the peak and an increased strain range. This can be attributed to the larger section size, which allows more extensive crack propagation and plastic deformation. However, when the cross-sectional aspect ratio further increases, the unevenness of the confinement effect limits the improvement in ductility. Overall, a smaller cross-sectional aspect ratio helps enhance peak stress, while a moderate increase in cross-sectional aspect ratio improves ductility. It is recommended that the cross-sectional aspect ratio should not exceed 1.7. In engineering design, the section shape should be selected based on the strength and ductility requirements, and stirrup arrangements or additional confinement measures should be optimized to balance load-bearing capacity and deformation ability.

### 3.4. Strain Analysis of Stirrup

**Figure 11:**
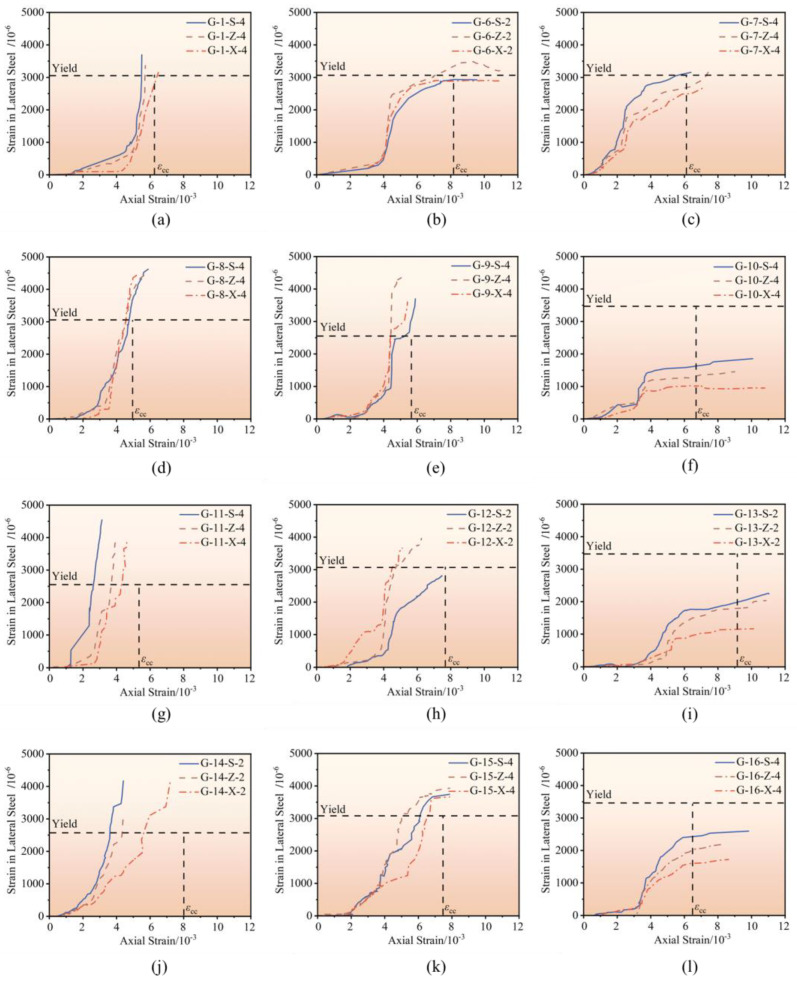
Measured strain curve of stirrup. (**a**) C-1; (**b**) C-6; (**c**) C-7; (**d**) C-8; (**e**) C-9; (**f**) C-10; (**g**) C-11; (**h**) C-12; (**i**) C-13; (**j**) C-14; (**k**) C-15; (**l**) C-16.

The measured stirrup strain curve for a typical specimen is shown in Figure 11. The horizontal axis represents the axial compressive strain of confined concrete, and the vertical axis represents the stirrup strain. The horizontal dashed line indicates the yield strain of the stirrup, while the vertical dashed line indicates the peak strain of the confined concrete. It can be observed that the stirrup stress varies significantly with axial strain, influenced by the stirrup strength. Before the specimen reaches its peak bearing capacity, the stirrup with a strength of 500 MPa has already yielded. Near the peak bearing capacity, the stirrup with a strength of 600 MPa partially enters the yielding state. In contrast, the stirrup with a strength of 700 MPa does not yield even after the specimen reaches its peak bearing capacity, showing higher strength reserves and sustained confinement capacity. High-strength stirrups provide effective lateral confinement at peak bearing capacity, delaying the damage progression in the concrete core and improving the specimen’s ductility. Therefore, it is recommended to use high-strength stirrups for high-strength concrete to ensure that the stirrup still has sufficient strength reserve at peak bearing capacity, thereby more effectively enhancing the specimen’s ductility.

## 4. Effective Lateral Binding of Stirrup

### 4.1. Effective Lateral Binding

**Figure 12:**
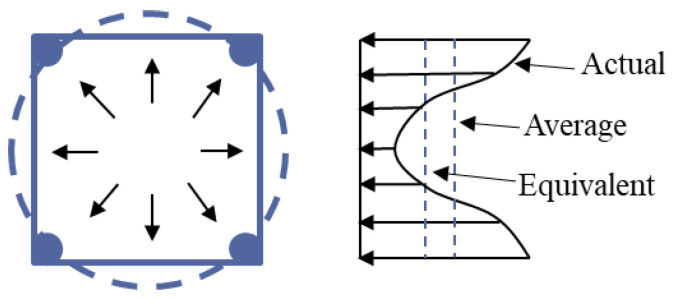
The distribution of passive constrained stress in the stirrup and its equivalent process diagram.

In a rectangular section, the core concrete expands laterally under pressure, and stirrups restrain it by applying a reaction force. However, due to the difference in the geometry and flexural stiffness of the stirrup along the section circumference, the binding force distribution is not uniform. At the corner of the stirrup, the bending stiffness is large, which can provide a strong lateral binding force. In the middle region of the stirrup edge, the flexural stiffness is low, and the binding force is significantly weakened, as shown in Figure 12. To simplify the calculation, it is assumed that the confining stress provided by the stirrup is uniformly distributed on all sides, and the average confining stress *f*_l_ can be obtained according to the force balance equation as follows:

(2) $$f_{l}=\frac{f_{\mathrm{lx}}d_{c}+f_{\mathrm{ly}}b_{c}}{b_{c}+d_{c}},$$

(3) $$f_{\mathrm{lx}}=\frac{A_{\mathrm{shx}}f_{\mathrm{sc}}}{sd_{c}},$$

(4) $$f_{\mathrm{ly}}=\frac{A_{\mathrm{shy}}f_{\mathrm{sc}}}{sb_{c}},$$

where *f*_sc_ is the stress of stirrup confining concrete under peak load; *f*_lx_ and *f*_ly_ are the lateral binding force of the stirrup in the direction of the long side and short side, respectively. *b*_c_ is the distance between the middle lines of the two stirrups on the long side of the cross-section, *d*_c_ is the distance between the middle lines of the two stirrups on the short side of the cross-section, and *s* is the longitudinal stirrup spacing.

**Figure 13:**
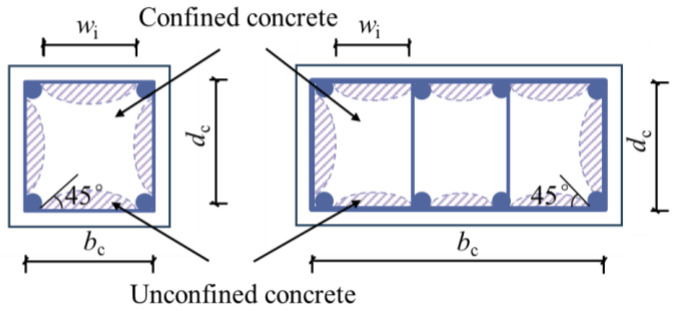
Schematic diagram of concrete area effectively confined by rectangular spiral stirrup cross-section.

**Figure 14:**
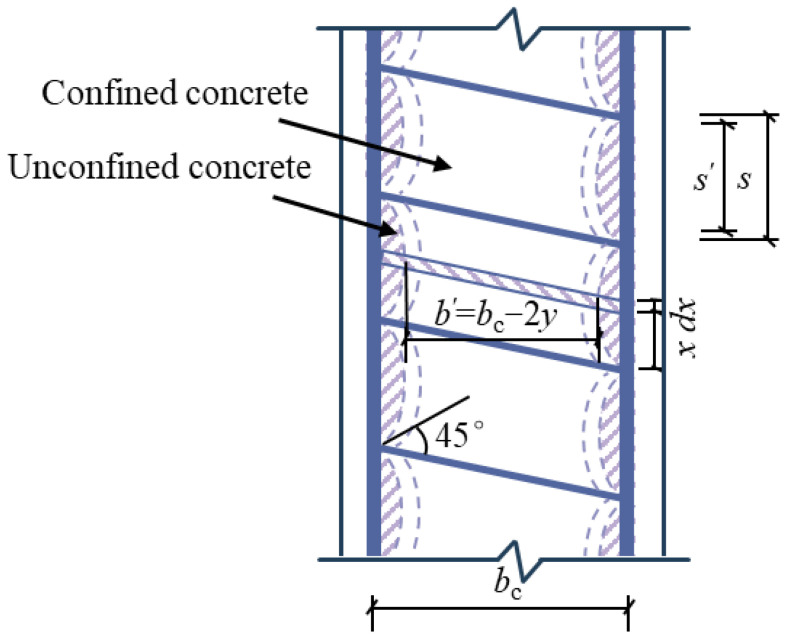
Schematic diagram of concrete area effectively restrained by rectangular spiral stirrup.

Due to the “arch effect”, the average lateral confinement stress only plays a full role in the effectively confined core concrete area shown in Figure 13 and Figure 14. Mander [9] modified the theory of effectively confined concrete in the core zone proposed by Sheikh and Uzumeri [26], redefined the concept of effectively confined core concrete area, and quantified the effective confined stress *f*_l’_ through the effective confinement coefficient *k*_e_. Then, the effective lateral confinement stress *f*_l’_ of the stirrup on the concrete in the core area is calculated as follows:

(5) $$f_{l}^{\prime }=k_{e}f_{l}$$

### 4.2. Effective Constraint Coefficient

Studies have shown that the area of the non-effective confinement region between two stirrups follows a parabolic distribution across the section. Assuming that the boundary curve of this non-effective confinement region is determined by the “arch effect,” it can be described using a quadratic parabola with an initial tangent slope of 45°. According to the Mander model, the effective confinement coefficient *k*_e_ can be expressed by the following Equation (6):

(6) $$k_{e}=\frac{A_{e}}{A_{\mathrm{cc}}},$$

(7) $$A_{e}=A_{\mathrm{ex}}I_{\mathrm{ey}},$$

where *A*_e_ is the area of concrete in the effective confinement area; *A*_cc_ is the area of the core concrete, equal to the core area surrounded by the stirrup minus the longitudinal reinforcement area. *A*_ex_ is related to the section shape of the column and the number and spacing of the longitudinal bars, expressed by the following Equation (8):

(8) $$A_{\mathrm{ex}}=b_{c}d_{c}-\sum_{i=1}^{n}\frac{{\left(W_{i}\right)}^{2}}{6},$$

where *W*_i_ indicates the net distance between adjacent longitudinal bars.

In rectangular spiral stirrup members, the core concrete is continuously constrained along the vertical direction due to the uninterrupted spiral arrangement of the stirrups. Unlike traditional stirrups, there is no section with weak confinement in the vertical direction. To quantify the longitudinal restraining effect of the rectangular spiral stirrup on the core concrete, this paper introduces the “coupling restraint coefficient” *I*_ey_, which characterizes the longitudinal and transverse coupling effect of the spiral stirrup. It is specifically defined as the ratio of the effective restraint volume *V*_e_ between the centerlines of adjacent stirrups to the core concrete volume *V*_cc_, which depends on the spacing and configuration of the stirrups, as follows:

(9) $$I_{\mathrm{ey}}=\frac{V_{e}}{V_{\mathrm{cc}}},$$

(10) $$V_{\mathrm{cc}}=sb_{c}d_{c}.$$

The quadratic parabola expression in the non-valid constraint region is

(11) $$y=-\frac{x^{2}}{s\prime }+\frac{s\prime }{4},$$

(12) $$b\prime =b_{c}-2y=b_{c}+\frac{2x^{2}}{s\prime }-\frac{s\prime }{2},$$

(13) $$d\prime =d_{c}-2y=d_{c}+\frac{2x^{2}}{s\prime }-\frac{s\prime }{2},$$

(14) $$A\left(x\right)=b\prime d\prime .$$

The concrete volume in the effective confinement zone between the net spacing of two adjacent stirrups (spacing *s’*) can be calculated by integrating the following:

(15) $$V_{e}\prime =\int_{-\frac{s^{\prime }}{2}}^{\frac{s^{\prime }}{2}}A\left(x\right)=\frac{{\left(s\prime \right)}^{3}}{4},$$

where *b’*, *d’*, and *A(x)* are the length, width, and area of the section of the effective constraint area.

The concrete volume *V*_e_ in the effective confinement zone between the center lines of two longitudinal adjacent stirrups (spacing *s*) can be expressed by Equation (16):

(16) $$V_{e}=V_{e}\prime +V_{c},$$

(17) $$V_{c}=b_{c}d_{c}\left(s-s\prime \right),$$

*V*_c_ represents the effective confined concrete volume within the stirrup’s diameter range (*s*-*s’*). By substituting Equations (16)–(18) into Equation (9), the following expression is obtained:

(18) $$I_{\mathrm{ey}}=\frac{V_{e}}{V_{\mathrm{cc}}}=\frac{{\left(s\prime \right)}^{3}}{4sb_{c}d_{c}}-\frac{s\prime }{s}+1.$$

By substituting Equations (8) and (18) into Equation (7), the *A*_e_ can be calculated as

(19) $$A_{e}=A_{\mathrm{ex}}I_{\mathrm{ey}}=\left[b_{c}d_{c}-\sum_{i=1}^{n}\frac{{\left(W_{i}\right)}^{2}}{6}\right]\left[\frac{{\left(s\prime \right)}^{3}}{4sb_{c}d_{c}}-\frac{s\prime }{s}+1\right].$$

By substituting Equations (14) and (10) into Equation (6), the *k*_e_ can be expressed as

(20) $$k_{e}=\frac{A_{e}}{A_{\mathrm{cc}}}=\frac{\left[1-\sum_{i=1}^{n}\frac{{\left(W_{i}\right)}^{2}}{6b_{c}d_{c}}\right]\left[\frac{{\left(s\prime \right)}^{3}}{4sb_{c}d_{c}}-\frac{s\prime }{s}+1\right]}{1-\rho_{\mathrm{cc}}}.$$

### 4.3. Stirrup Stress

For HRSS-confined steel fiber reinforced concrete, high-strength stirrups may not yield at peak strength. Therefore, when calculating the stirrup stress corresponding to the peak load of confined concrete, it should be estimated based on the actual stress rather than simply assuming it equals the yield strength. Otherwise, there is a risk of overestimating the stirrup’s confinement effect. The main factors influencing the actual stirrup stress at peak load include the yield strength of the stirrup, stirrup spacing, section size, and concrete strength. For specimens where the stirrup did not yield when the confined concrete reached its compressive strength in the test, the stirrup tensile stress *f*_sc_ at the peak compressive stress of the confined concrete can be fitted as follows:

(21) $$f_{\mathrm{sc}}=0.12{\left(\frac{E_{s}k_{e}\rho_{v}}{f_{c0}^{\prime }}\right)}^{1.22}+52,$$

where *E*_s_ is the elastic modulus and *ρ*_v_ is the volume stirrup ratio of the column. *f*_c0_ is the peak stress of unconfined concrete, and *k*_e_ is the effective confinement coefficient.

**Figure 15:**
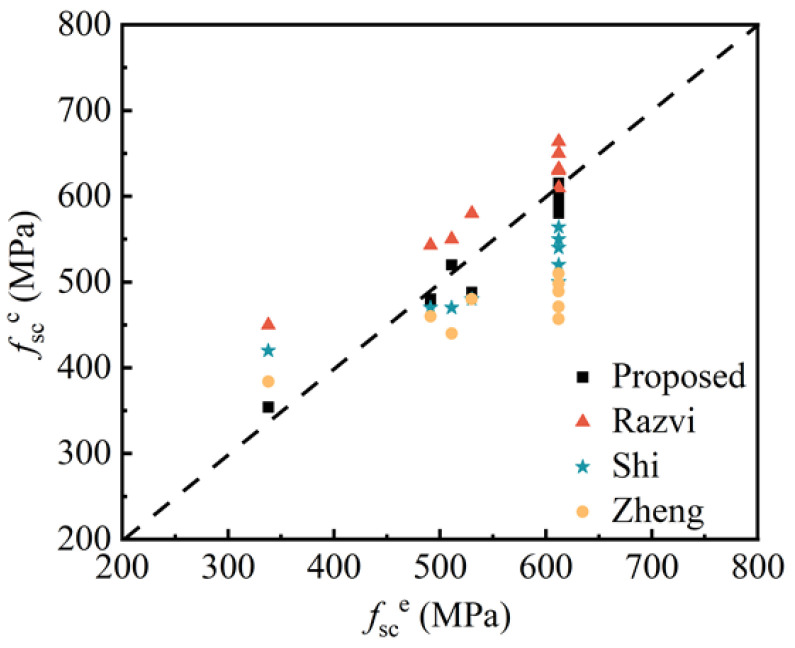
Comparison between calculated and tested values of stirrup stress.

As shown in Table 7, the calculation formulas for stirrup tensile stress at peak compressive stress of confined concrete proposed by different scholars are listed. The comparison between the experimental stirrup stress *f_s_*_c_^e^ and the calculated stirrup stress *f_s_*_c_^c^ at peak bearing capacity for each specimen in the axial compression tests is shown in Figure 15, where *f_s_*_c_^e^ is taken as the maximum measured value from each stirrup measurement point. The comparison results indicate that the calculation values from the formula proposed by Razvi et al. [27] are consistently higher than the experimental values across different stirrup stress levels. The formula proposed by Shi et al. [28] overestimates the stirrup stress at low levels but underestimates it at high levels. The formula by Zheng et al. [29] yields good agreement with the experimental values at low stirrup stress, but the calculation values are lower than the experimental values at high stirrup stress. However, the calculation values from the fitting formula presented in this study match the experimental values better, more accurately reflecting the actual distribution of stirrup stress.

## 5. Constitutive Model

### 5.1. Peak Parameter

Through the regression analysis of the test results, the formulas for calculating the peak strength, peak strain, and ultimate strain of steel fiber reinforced concrete confined by rectangular spiral stirrup can be obtained:

(22) $$\frac{f_{\mathrm{cc}}}{f_{c0}}=1+8.86{\left(\frac{f_{\mathrm{lx}}^{\prime }}{f_{c0}}\right)}^{1.99}+7.72{\left(\frac{f_{\mathrm{ly}}^{\prime }}{f_{c0}}\right)}^{0.66},$$

(23) $$\frac{\varepsilon_{\mathrm{cc}}}{\varepsilon_{c0}}=1+5.42{\left(\frac{f_{\mathrm{lx}}^{\prime }}{f_{c0}}\right)}^{8.85}+9.57{\left(\frac{f_{\mathrm{ly}}^{\prime }}{f_{c0}}\right)}^{0.07}.$$

In the formula, *f*_cc_ and *f*_c0_, *ε*_cc_ and *ε*_c0_ are the peak strength and peak strain of confined and unconfined concrete, respectively.

**Figure 16:**
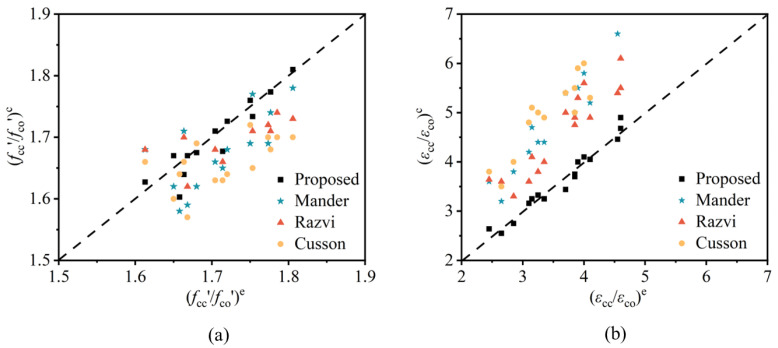
Comparison of calculated stress and strain of stirrup with experimental values. (**a**) Stirrup stress; (**b**) stirrup strain.

The peak stress and strain calculation models for confined concrete proposed by different scholars are listed in Table 8. Figure 16a,b shows the comparison analysis of the peak stress enhancement ratio (*f_cc_*/*f*_c0_)^e^ and its calculated value (*f_cc_*/*f*_c0_)^c^, and the peak strain enhancement ratio (*ε*_cc_/*ε*_c0_)^e^ and its calculated value (*ε*_cc_/*ε*_c0_)^c^, respectively. The points represent the relationship between the actual observed data of each specimen and the model’s predicted values. The comparison results indicate that the other models generally underestimate the peak stress of confined steel fiber reinforced concrete, overestimate the peak strain, and exhibit significant dispersion. In contrast, the peak stress and strain predicted by the model suggested in this study show a good agreement with the experimental values, accurately predicting the mechanical behavior of confined steel fiber reinforced concrete.

### 5.2. Descending Stage Parameter

According to the test results, the compressive strain corresponding to 85% of the peak compressive stress and 65% of the peak compressive stress in the descending section of the confined concrete are fitted. The regression equations of *ε*_85_/*ε*_c0_ and *ε*_65_/*ε*_c0_ obtained by nonlinear fitting are as follows:

(24) $$\frac{\varepsilon_{85}}{\varepsilon_{c0}}=1+0.20{\left(\frac{f_{\mathrm{lx}}^{\prime }}{f_{c0}^{\prime }}\right)}^{9.30}+0.99{\left(\frac{f_{\mathrm{ly}}^{\prime }}{f_{c0}^{\prime }}\right)}^{0.20},$$

(25) $$\frac{\varepsilon_{65}}{\varepsilon_{c0}}=1+7.76{\left(\frac{f_{\mathrm{lx}}^{\prime }}{f_{c0}^{\prime }}\right)}^{18.23}+7.86{\left(\frac{f_{\mathrm{ly}}^{\prime }}{f_{c0}^{\prime }}\right)}^{0.55}.$$

In the formula, *ε*_85_ and *ε*_65_ are the corresponding compressive strains when the compressive stress of the restrained concrete drops to 85% and 65% of the peak compressive stress, respectively.

### 5.3. Stress-Strain Full Curve Model

On the basis of the experiments, this paper proposes the stress–strain full curve of HRSS-confined steel fiber reinforced concrete. The expression forms of the Mander model [9] and the Fafitis and Shah model [30] are used in the rising and falling sections, respectively. The model proposed by Mander is widely recognized for its accuracy in describing the stress–strain characteristics of confined concrete, particularly in the ascending part. It effectively reflects the enhancement of strength and ductility due to the confining effect, making it suitable for the initial loading phase. The model proposed by Fafitis and Shah provides a reliable framework for characterizing the descending portion of the stress–strain curve, reflecting the softening behavior of concrete after it reaches its peak stress. The expression of the full curve is as follows:

(26) $${f_{c}=f_{\mathrm{cc}}{\left[\frac{\gamma \left(\varepsilon_{c}/\varepsilon_{\mathrm{cc}}\right)}{\gamma -1+{\left(\varepsilon_{c}/\varepsilon_{\mathrm{cc}}\right)}^{\gamma }}\right]}_{\ldots }}_{\left(\varepsilon_{c}\leq \varepsilon_{\mathrm{cc}},\right)}$$

(27) $$\gamma =\frac{E_{c}}{E_{c}-(f_{\mathrm{cc}}/\varepsilon_{\mathrm{cc}})},$$

(28) $$f_{c}=f_{\mathrm{cc}}\cdot \exp\left[\alpha {\left(\varepsilon_{c}/\varepsilon_{\mathrm{cc}}\right)}^{\beta }\right]\ldots \left(\varepsilon_{c}\geq \varepsilon_{\mathrm{cc}}\right),$$

where *E*_c_ is the tangential modulus of concrete and *γ* is the coefficient controlling the initial stiffness and the ascending section of the curve. *α* and *β* are the factors controlling the slope and convex and concave of the descending section of the curve, respectively.

The characteristic parameters (*ε*_85_, 0.85 *f*_cc_) and (*ε*_65_, 0.65 *f*_c_) of the descending section that has been fitted above are substituted into Equation (28), and the calculation formulas of parameters *α* and *β* are calculated and simplified as follows:

(29) $$\alpha =\frac{\ln0.85}{{\left(\varepsilon_{85}-\varepsilon_{\mathrm{cc}}\right)}^{\beta }},$$

(30) $$\beta =\frac{\ln\left(\frac{\ln\left(0.85\right)}{\ln\left(0.65\right)}\right)}{\ln\left(\frac{\varepsilon_{85}-\varepsilon_{\mathrm{cc}}}{\varepsilon_{65}-\varepsilon_{\mathrm{cc}}}\right)}$$

**Figure 17:**
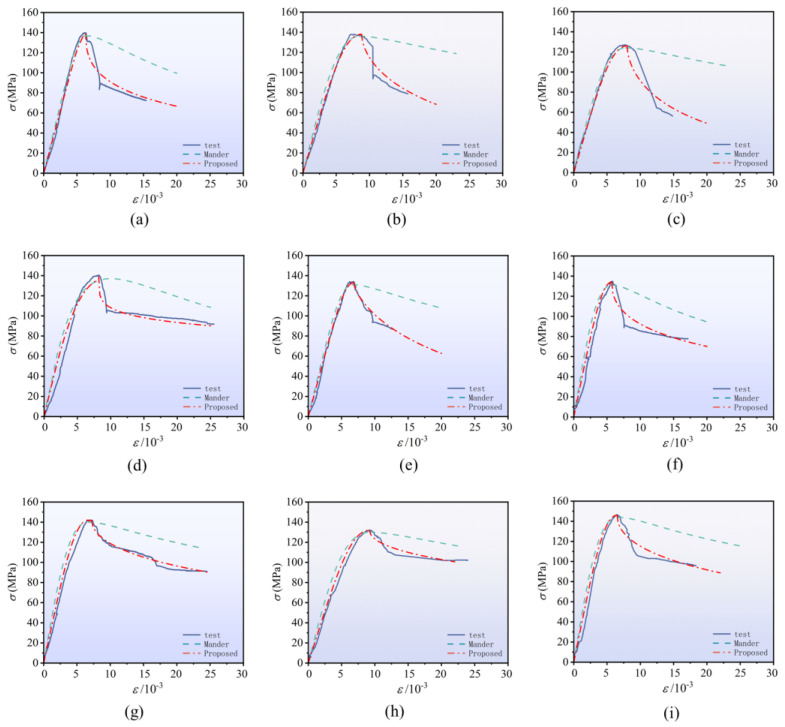
Comparison of stress–strain curves of typical specimens. (**a**) C-1; (**b**) C-4; (**c**) C-5; (**d**) C-6; (**e**) C-7; (**f**) C-9; (**g**) C-10; (**h**) C-13; (**i**) C-16.

As shown in Figure 17, for the comparison between the calculated curve and the measured curve of typical specimens in this paper, the Mander model generally underestimated the peak strength, there was a deviation at the peak strain position, and the prediction of the falling section was relatively gentle, which failed to fully reflect the actual deformation behavior of specimens. In this study, it is proposed that the model is in agreement with the test data in predicting the peak strength and peak strain and can accurately reflect the ductility characteristics of the specimen and the changing trend of the descending section of the stress–strain curve.

## 6. Conclusions

This study designs and conducts axial compression tests on 16 HRSS confined steel fiber reinforced concrete columns, analyzing the effects of stirrup spacing, stirrup strength, concrete strength, and section aspect ratio on the failure modes, stress–strain curves, stirrup stress development, as well as bearing capacity and ductility. Based on the experimental data, a “coupling restraint coefficient” is proposed, and a calculation method for the HRSS confined steel fiber reinforced concrete constitutive model is established, considering the coupled effect of effective confinement forces in different directions. The main conclusions are as follows:

(1) The incorporation of steel fibers and the use of high-strength rectangular spiral stirrups significantly improved the failure characteristics of SFRC specimens. Due to the bridging effect of steel fibers, the specimens exhibited a more pronounced ductile failure mode, with cracks appearing in the SFRC cover but without complete spalling;

(2) When the stirrup spacing is less than one-third of the shortest side length, the section aspect ratio is less than 1.67, and high-strength concrete is paired with high-strength stirrups, the rectangular spiral stirrup-confined steel fiber reinforced concrete specimens exhibit better strength and ductility performance;

(3) The lateral confinement force provided by stirrups is an important factor influencing the mechanical performance of confined concrete. Under the confinement of high-strength rectangular spiral stirrups, the stirrup strain may not necessarily yield when the concrete reaches its compressive strength. Based on theoretical analysis and experimental data, a stirrup stress calculation method is proposed that can accurately describe the stirrup stress variation at the peak stress of confined concrete, providing a theoretical basis for the design of HRSS confined steel fiber reinforced concrete columns;

(4) A “coupling restraint coefficient” is proposed, and a constitutive model for HRSS confined steel fiber reinforced concrete is developed, considering the coupled effect of effective confinement forces in different directions. The model introduces effective confinement coefficients, peak parameters, and descending parameters, which can accurately describe the mechanical behavior of concrete under different confinement levels. The model shows good agreement with experimental data and can serve as a reference for the theoretical analysis and engineering design of such components;

(5) The findings of this study provide new theoretical insights for the design of steel fiber-reinforced concrete columns and offer valuable references for practical structural design. The application of high-strength rectangular spiral stirrups significantly enhances the compressive strength and ductility of the structure, making it particularly suitable for areas prone to earthquakes and high-load conditions. Therefore, the research results provide solid theoretical support and engineering guidance for the seismic design of related structures.

## Figures and Tables

**Table 1:** Detailed design parameters of the specimen.

Specimens	*b* × *d* × *h*/mm	Grade of Concrete	Transverse Reinforcement			Longitudinal Reinforcement
			Type	*s*/mm	*ρ*_v_/%	
C-1	150 × 150 × 450	C100	600	40	1.1	4 C 16
C-2	170 × 170 × 510	C100	600	35	1.1	4 C 16
C-3	200 × 200 × 600	C100	600	30	1.1	4 C 16
C-4	250 × 150 × 750	C100	600	40	1.2	8 C 16
C-5	300 × 150 × 900	C100	600	40	1.1	8 C 16
C-6	150 × 150 × 450	C100	600	20	2.1	4 C 16
C-7	150 × 150 × 450	C100	600	60	0.7	4 C 16
C-8	150 × 150 × 450	C100	600	80	0.5	4 C 16
C-9	150 × 150 × 450	C100	500	40	1.1	4 C 16
C-10	150 × 150 × 450	C100	700	40	1.1	4 C 16
C-11	150 × 150 × 450	C80	500	40	1.1	4 C 16
C-12	150 × 150 × 450	C80	600	40	1.1	4 C 16
C-13	150 × 150 × 450	C80	700	40	1.1	4 C 16
C-14	150 × 150 × 450	C110	500	40	1.1	4 C 16
C-15	150 × 150 × 450	C110	600	40	1.1	4 C 16
C-16	150 × 150 × 450	C110	700	40	1.1	4 C 16

Note: *b*, *d*, and *h* stand for length, width, and height, respectively; *s* is the stirrup spacing; *ρ*_v_ is the volume stirrup ratio.

**Table 2:** Detailed mix ratio of steel fiber reinforced concrete.

Intensity	Raw Material	P.O42.5 Cement	Silica Fume	Sand	Water	Water Reducing Agent	Steel Fiber
C80	content (kg·m^−3^)	1032.97	258.24	697.24	361.55	11.61	78.5(1%)
C100		1074.09	268.64	725	281.82	9.55	78.5(1%)
C110		990.157	330.06	712.91	316.85	6.61	78.5(1%)

**Table 3:** The performance index of steel fibers.

Length/mm	Diameter/mm	Slenderness Ratio	Mass Density /g/cm3	Tensile Strength/MPa
14	0.2	65	7.85	2850

**Table 4:** Basic mechanical properties of concrete.

Design Concrete Grade	*f*_ck_/(MPa)			*f*_cu_/(MPa)	Standard Deviation	*f*_c0_/(MPa)
	Test Block 1	Test Block 2	Test Block 3			
C80	80.74	79.81	83.93	81.49	1.77	65.19
C100	96.88	98.35	100.44	98.56	1.46	78.84
C110	109.27	112.16	111.18	110.87	1.20	88.69

**Table 5:** Mechanical properties of reinforcement.

Grade of Reinforcement		*d*/mm	*f*_y_/MPa	*E*_s_/MPa	*ε*_y_/10^−6^
HRB400		16	440.3	2.0 × 105	2202
High-strength steel wire	Grade 500	5	511.1	2.0 × 105	2555
	Grade 600	5	611.5	2.0 × 105	3058
	Grade 700	5	691.4	2.0 × 105	3457

**Table 6:** The main test results of the specimen.

Specimens	*N*/kN	*f*_c0_/MPa	*f*_cc_/MPa	*ε*_cc_	*ε*_0.85_	*ε*_0.65_	*f*_cc_/*f*_c0_	*ε*_0.85_/*ε*_cc_
C-1	2897.06	78.85	140.07	0.0063	0.0073	0.0084	1.78	1.16
C-2	3552.20	78.85	131.14	0.0118	0.0136	0.0203	1.66	1.53
C-3	4420.08	78.85	113.57	0.0091	0.0102	0.0132	1.44	1.12
C-4	5009.71	78.85	138.21	0.0077	0.0105	0.0124	1.75	1.36
C-5	5616.68	78.85	127.15	0.0078	0.0100	0.0123	1.61	1.28
C-6	2906.17	78.85	140.74	0.0082	0.0092	0.0255	1.79	1.12
C-7	2819.43	78.85	134.36	0.0062	0.0082	0.0126	1.70	1.32
C-8	2780.68	78.85	131.51	0.0049	0.0061	0.0071	1.67	1.26
C-9	2829.90	78.85	135.13	0.0057	0.0070	0.0092	1.71	1.23
C-10	2928.20	78.85	142.36	0.0067	0.0092	0.0205	1.81	1.37
C-11	2618.58	65.20	127.72	0.0053	0.0084	0.0152	1.96	1.59
C-12	2453.94	65.20	115.61	0.0077	0.0095	0.0165	1.77	1.23
C-13	2682.76	65.20	132.44	0.0092	0.0118	0.0244	2.03	1.11
C-14	2891.74	88.70	133.81	0.0080	0.0104	0.0184	1.51	1.28
C-15	2756.32	88.70	123.85	0.0074	0.0084	0.0095	1.40	1.30
C-16	3071.48	88.70	147.03	0.0065	0.0082	0.0184	1.66	1.26

**Table 7:** Formula for calculating stirrup stress under confined concrete Peak compressive stress.

Stirrup Tensile Stress Calculation Formula	Reference
$f_{\mathrm{sc}}=E_{s}\left(0.0025+0.04\sqrt[{3}]{\frac{k_{e}\rho_{v}}{f_{c0}}}\right)\leq f_{\mathrm{yv}}$	Razvi et al. [27]
$f_{\mathrm{sc}}=0.05E_{s}k_{e}\rho_{v}\sqrt{\frac{f_{\mathrm{yv}}}{f_{c0}}}+180\leq f_{\mathrm{yv}}$	Shi et al. [28]
$f_{\mathrm{sc}}=E_{s}\left(30.9k_{e}\rho_{v}^{3}\sqrt[{3}]{\frac{E_{s}}{f_{c0}}}+0.00015\right)\leq f_{\mathrm{yv}}$	Zheng et al. [29]
$f_{\mathrm{sc}}=0.12{\left(\frac{E_{s}k_{e}\rho_{v}}{f_{c0}^{\prime }}\right)}^{1.22}+52\leq f_{\mathrm{yv}}$	this work

**Table 8:** Calculation model of peak stress and strain of concrete with different confinement.

*f*_cc’_	*ε*_cc_	Reference
$f_{\mathrm{cc}}^{\prime }=f_{c0}^{\prime }\left(-1.254+2.254\sqrt{1+\frac{7.94f_{l}^{\prime }}{f_{c0}^{\prime }}}-2\frac{f_{l}^{\prime }}{f_{c0}^{\prime }}\right)$	$\varepsilon_{\mathrm{cc}}=\varepsilon_{c0}\left[1+5\left(\frac{f_{\mathrm{cc}}^{\prime }}{f_{c0}^{\prime }}-1\right)\right]$	Mander [9]
$f_{\mathrm{cc}}^{\prime }=f_{c0}^{\prime }\left[1.0+2.1{\left(\frac{f_{l}^{\prime }}{f_{c0}^{\prime }}\right)}^{0.7}\right]$	$\varepsilon_{\mathrm{cc}}=\varepsilon_{c0}\left[1+0.21{\left(\frac{f_{l}^{\prime }}{f_{c0}^{\prime }}\right)}^{1.7}\right]$	Cusson [12]
$f_{\mathrm{cc}}^{\prime }=f_{c0}^{\prime }+4.1f_{l}$	$\varepsilon_{\mathrm{cc}}=\varepsilon_{c0}\left(1+5k_{3}K\right)$	Razvi et al. [27]
$\frac{f_{\mathrm{cc}}^{\prime }}{f_{c0}^{\prime }}=1+8.86{\left(\frac{f_{\mathrm{lx}}^{\prime }}{f_{c0}^{\prime }}\right)}^{1.99}+7.72{\left(\frac{f_{\mathrm{ly}}^{\prime }}{f_{c0}^{\prime }}\right)}^{0.66}$	$\frac{\varepsilon_{\mathrm{cc}}}{\varepsilon_{c0}}=1+5.42{\left(\frac{f_{\mathrm{lx}}^{\prime }}{f_{c0}^{\prime }}\right)}^{8.85}+9.57{\left(\frac{f_{\mathrm{ly}}^{\prime }}{f_{c0}^{\prime }}\right)}^{0.07}$	this work

## Data Availability

The original contributions presented in this study are included in the article. Further inquiries can be directed to the corresponding authors.
